# HIV-1 TAR element is processed by Dicer to yield a viral micro-RNA involved in chromatin remodeling of the viral LTR

**DOI:** 10.1186/1471-2199-8-63

**Published:** 2007-07-30

**Authors:** Zachary Klase, Prachee Kale, Rafael Winograd, Madhur V Gupta, Mohammad Heydarian, Reem Berro, Timothy McCaffrey, Fatah Kashanchi

**Affiliations:** 1 Immunology, Microbiology and Tropical Medicine program, The George Washington University School of Medicine, Washington, District of Columbia 20037, USA; 2 Department of Biochemistry and Molecular Biology, The George Washington University School of Medicine, Washington, District of Columbia 20037, USA; 3 Genetics program, The George Washington University School of Medicine, Washington, District of Columbia 20037, USA; 4 The Institute for Genomic Research, TIGR, Rockville, Maryland 20850, USA

## Abstract

**Background:**

RNA interference (RNAi) is a regulatory mechanism conserved in higher eukaryotes. The RNAi pathway generates small interfering RNA (siRNA) or micro RNA (miRNA) from either long double stranded stretches of RNA or RNA hairpins, respectively. The siRNA or miRNA then guides an effector complex to a homologous sequence of mRNA and regulates suppression of gene expression through one of several mechanisms. The suppression of gene expression through these mechanisms serves to regulate endogenous gene expression and protect the cell from foreign nucleic acids. There is growing evidence that many viruses have developed in the context of RNAi and express either a suppressor of RNAi or their own viral miRNA.

**Results:**

In this study we investigated the possibility that the HIV-1 TAR element, a hairpin structure of ~50 nucleotides found at the 5' end of the HIV viral mRNA, is recognized by the RNAi machinery and processed to yield a viral miRNA. We show that the protein Dicer, the enzyme responsible for cleaving miRNA and siRNA from longer RNA sequences, is expressed in CD4+ T-cells. Interestingly, the level of expression of Dicer in monocytes is sub-optimal, suggesting a possible role for RNAi in maintaining latency in T-cells. Using a biotin labeled TAR element we demonstrate that Dicer binds to this structure. We show that recombinant Dicer is capable of cleaving the TAR element in vitro and that TAR derived miRNA is present in HIV-1 infected cell lines and primary T-cell blasts. Finally, we show that a TAR derived miRNA is capable of regulating viral gene expression and may be involved in repressing gene expression through transcriptional silencing.

**Conclusion:**

HIV-1 TAR element is processed by the Dicer enzyme to create a viral miRNA. This viral miRNA is detectable in infected cells and appears to contribute to viral latency.

## Background

RNA interference (RNAi) is a regulatory mechanism found in plants, nematodes, protozoan, *Drosophila*, and mammalian cells [1-3]. Double stranded RNA is recognized by the RNAi machinery and is processed into small, 21 nucleotide small-interfering RNA (siRNA) which are capable of suppressing gene expression. Exogenously introduced dsRNA is recognized by the cellular ribonuclease III enzyme Dicer and cleaved into 21 nucleotide segments. Endogenously expressed RNA can be involved in RNAi through a slightly different pathway involving Drosha mediated cleavage of RNA stemloops in the nucleus, followed by exportation to the cytoplasm by Exportin-5, and finally cleavage by Dicer to generate a small RNA duplex, called microRNA (miRNA) [4-9].

One strand of the miRNA duplex is incorporated into one of two Argonaute containing effector complexes which silence gene expression through two different mechanisms. In the first, the small RNA associates with the RNA-induced silencing complex (RISC) and guides the complex to a complementary sequence of mRNA where a member of the Argonaute family of proteins cleaves the target mRNA [1-3,10]. Alternatively, the miRNA may guide the RISC complex to a complementary, but not perfectly matching, region in the 3'UTR of the mRNA. This association inhibits protein translation without degrading the target mRNA [2,11,12]. Alternatively the RNA can associate with the RNA-induced initiation of transcriptional silencing (RITS) complex. The miRNA guides this complex to a complementary region of chromosomal DNA and recruits factors that modify the chromatin structure and induce transcriptional silencing [13-16].

The two pathways of RNAi likely serve several purposes. Recognition of foreign RNA and subsequent processing by RISC may serve as a defense mechanism against viral infection [17]. RNAi may serve as a means for cells to maintain specific chromosomal architecture and repress transcription of retro-transposons [13,18,19]. Additionally, it has been discovered that miRNA generated by the cell is important for embryonic development and RNAi may serve as a broad regulator of gene expression [20-23].

The structures of many viral RNAs likely provide appropriate targets for the RNAi machinery. Several viruses have already been identified that yield small RNAs after Dicer processing, including; human cytomegalovirus, human herpesevirus 8, Epstein Barr virus and peach latent mosaic viroid [24-26]. Another group has reported that a miRNA is produced from the Nef-RNA of HIV-1 [27]. Recent computer modeling has also predicted the existence of up to five structures in HIV-1 RNA that may be processed by Dicer to yield miRNA [28,29]. Additionally, it has been shown that a region within the Env gene contains a stemloop structure that is potentially acted upon by Dicer and the components of RNAi [30].

If HIV-1 does produce RNA structures that are recognized and cleaved by Dicer, then this could give rise to several interesting possibilities that would be consistent with the discovery that HIV downregulates many cellular genes [31-33]. A viral miRNA generated from full-length, doubly spliced or singly spliced HIV-1 transcripts could potentially inhibit viral replication or down regulate cellular gene expression. Dicer cleavage products of viral RNA may target the RISC complex to degrade viral mRNA or block translation of viral proteins, through sequence complementarity. HIV miRNAs may associate with the RITS complex and cause chromatin remodeling of the viral genome, leading to transcriptional repression. Indeed, the integrated HIV genome is associated with chromatin remodeling complexes and recruitment of proteins with histone acetyl-transferase activity that are needed for proper activation of the virus [34-36]. And finally, it may be possible that these miRNAs do not target viral genes, but instead regulate the expression of cellular proteins [28]. HIV infection has already been shown to alter host gene expression through other mechanisms, such as the viral proteins Tat, Nef and Vpr [31-33].

Therefore, suppression of viral replication or alteration of host cell gene expression by viral miRNA may be a possible mechanism involved in latency. In latent cells there is a low level of transcription and very little production of viral proteins [37,38]. Due to this lack of transcription there is a very low level of Tat protein available to recruit various Cyclin/cdks and chromatin-remodelling factors needed for activated transcription. As a result, latent cells may produce a high level of short, abortive RNA transcripts only 50–100 nt in length, that contain the HIV TAR stemloop. To address this possibility, the Peterlin lab isolated PBMCs from HIV-1 infected patients, analyzed the transcriptional profile using RT-PCR, and found that in some patients only short transcripts of less than 97 nucleotides were detectable [39,40]. Also, Lassen and Siliciano purified resting CD4+ T-cells from HIV-1 patients and showed the presence of short transcripts in both viremic and non-viremic patients, with short transcripts being more abundant than longer processive transcripts in the non-viremic patients [41]. The HIV-1 TAR element is similar to known dicer substrates, as it is an imperfect stemloop of approximately 50 nucleotides [42]. Additionally, computer modeling has predicted TAR to be one of five structures in HIV that is possibly processed by Dicer [28]. As these TAR-containing short transcripts are the only HIV RNA produced in appreciable quantities during latency it is possible that miRNAs generated from TAR may work to suppress viral gene expression or alter host-cell proteins levels, and in doing so, maintain the latent state.

Further support of a role for TAR in RNAi stems from recent findings that identify TAR-RNA Binding Protein (TRBP) as the human homologue of the *Drosophila *Loquacious protein [43,44]. Loquacious and TRBP are Dicer binding partners that are required for efficient loading of the miRNA into the RISC complex [43]. Interestingly, TRBP was discovered over a decade ago through its association with the TAR element and plays a role in transactivation and inhibition of interferon induced PKR [45-48]. That other components of the RNAi pathway, such as TRBP, can be found associated with the TAR element is striking evidence that TAR may be processed to yield miRNA.

Here, we show evidence that the short transcripts in latently infected cells may lead to production of a viral miRNA. We show that Dicer is expressed in CD4+ T-cell lines and in primary cells isolated from healthy donors. Interestingly, Dicer levels monocytic cell lines and primary cell was found to be sub-optimal. Using a biotin labeled TAR RNA we show that Dicer, from whole cell extracts, is capable of binding the TAR structure. This interaction is specific and can be blocked by addition of an unlabeled competitor but not by a mutant TAR. *In vitro *transcribed TAR is cleaved by recombinant Dicer to yield a 21 nucleotide RNA duplex. Analysis of a selection of TAR mutants reveals that changes in the sequence of the TAR structure have little effect on Dicer cleavage. Specifically TAR mutants that are deficient for the Tat binding site or the terminal loop are still cleaved by Dicer. Only one mutant, which had a shortened stem, was incapable of being processed by Dicer. Additionally, we show evidence for existence of a HIV-1 TAR derived miRNA in HIV-1 infected cells through the use of an RNase protection assay (RPA). This miRNA may be incorporated into the RISC or the RITS complex and suppress either viral or cellular gene expression.

## Results

### HIV-1 target cells express Dicer

**Figure 1:**
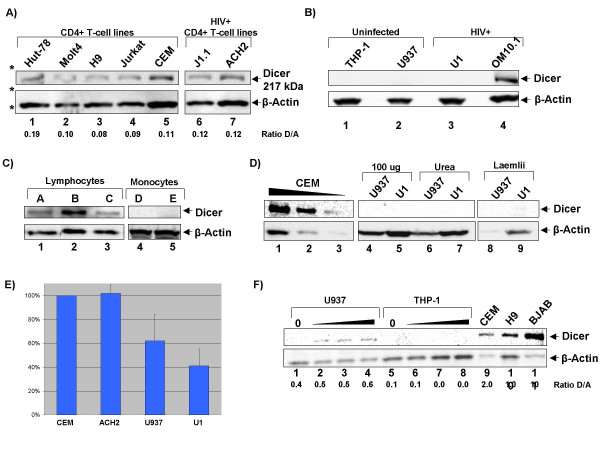
**Dicer expression in HIV-1 infected and target cell-lines**. **A) **Thirty micrograms of whole cell extract from CD4+ T-cell lines (lane 1 – Hut78, lane 2 – Molt 4, lane 3 – H9, lane 4 – Jurkat, lane 5 – CEM) and HIV-1 infected CD4+ T-cell lines (lane 6 – J1.1, lane 7 – ACH2) was resolved by SDS-PAGE and western blotted for the presence of Dicer and β-actin. Asterisks indicate the position of the 220, 45 and 30 kDa mass standards. Densitometry was performed and the ratio of Dicer to β-actin was determined and is shown below the western blot images. **B) **Thirty micrograms of whole cell extract from monocytes (lane 1 – THP-1), pro-monocytes (lane 2 – U937), HIV-1 infected pro-monocytes (lane 3 – U1) and HIV-1 infect pro-myelocytes (lane 4 – OM10.1) was resolved by SDS-PAGE and western blotted for the presence of Dicer and β-actin. **C) **Monocytes and lymphocytes were isolated from three normal donors (designated A, B, C, D and E) as described in materials and methods. Five micrograms of whole cell extract from lymphocytes and 20 micrograms from monocytes was resolved by SDS-PAGE and western blotted for the presence of Dicer. **D) **Decreasing amounts of CEM extract were western blotted for Dicer Lanes 1 – 20 micrograms, 2 – 10 microgramsm 3 – 5 micrograms. 100 micrograms of U937 and U1 extract were blotted (lanes 4 and 5). Urea buffer was used to extract proteins from U937 and U1. Fifty micrograms of extract was western blotted for Dicer (lanes 6 and 7). U937 and U1 cells were dissolved in Laemlii buffer for 2 minutes at 95° and loaded directly onto the gel (lanes 8 and 9) **E) **Quantitative RT-PCR analysis was performed to examine the level of Dicer expression in CEM, ACH2, U937 and U1. Relative expression values were obtained by normalization to actin. **F) **U937 and THP-1 were cultured in the presence of 0, 10, 20 or 100 nM PMA for 48 hours to induce differentiation. Twenty micrograms of whole cell extract from U937, THP-1, CEM, H9 and BJAB were resolved by SDS-PAGE and western blotted for Dicer. Densitometry was performed as in panel A.

As Dicer is the major catalytic engine that generates miRNA through the cleavage of dsRNA, we sought to determine if cell lines relevant to HIV-1 infection also expressed Dicer. We tested cell extracts from CD4+ T-cell lines (Hut78, Molt4, H9, Jurkat, CEM) and HIV-1 infected CD4+ T-cell lines (J1.1, ACH2). Dicer expression was detected in all of the T-cell types tested (Fig 1A). Normalization to β-actin (Fig 1A, lower panel and ratios listed below) revealed that the T-cell lines express similar levels of Dicer, with the exception of Hut-78 cells that appear to express about twice as much Dicer as the others (Compare lane 1 to lanes 2–7). Interestingly, HIV-1 infection does not appear to significantly influence Dicer expression based on our analysis of Jurkat and CEM lines as compared to their infected counterparts (compare lane 4 to 6 and 5 to 7). This suggests that Dicer is present in uninfected and HIV-1 infected cells. That Dicer is present suggests that miRNA may be generated by these cell types. These results confirm previous studies that have shown production of miRNA, correlating with active Drosha and Dicer enzymes, and an active RNAi system in T-lymphocytes [22,23,49,50].

We next sought to determine if Dicer is present in cells of myeloid lineage. Monocytes (THP-1), pro-monocytes (U937), HIV-1 infected pro-monocytes (U1) and infected pro-myelocytes (OM10.1) were tested for Dicer expression (Fig 1B). With the exception of OM10.1 pro-myeloctyes, none of the myeloid cells expressed levels of Dicer visible by our western blots (Fig 1B, compare lanes 1–3 to 4). OM10.1 are a derivative of HL-60 that have survived acute infection with HIV-1 [51]. HL-60 are a pro-myeloid cell line capable of differentiating into basophils, neutrophils, eosinophils and monocytes. Butyric acid can specifically promote differentiation of these cells into neutrophils [52,53]. The infection of OM10.1 represents an artificial situation that allowed the study of CD4 levels in response to infection [51]. That western blot could not detect Dicer expression in monocytes, as compared to T-cells, is interesting as CD4+ T-cells support latent infection in HIV, but monocytes and macrophages do not [38,54]. The detectable Dicer expression in the pro-myeloid OM10.1, a pro-genitor of monocytes, cells is likely related to Dicer's role in differentiation and development. Previous studies have shown that the components of RNAi are necessary for control of development and maintenance of pro-genitor cells [11,20,22,43]. Indeed, analysis of miRNA expression patterns in differentiating HL-60 cells suggest an involvement of RNAi in differentiation [55].

To verify these findings in a more relevant set of cells we isolated PBMCs from healthy donors (Designated A, B, C, D and E). After separation of PBMCs from whole blood using ficoll, monocytes were separated from lymphocytes (CD4+ T-cells, CD8+ T-cells and B-cells) by plastic adherence. The lymphocytes, in the suspension fraction, were removed from the plate, washed with PBS and lysed. Monocytes that were adhered to the plate were washed once with PBS to remove any remaining lymphocytes, scraped from the plate and lysed. Twenty micrograms of whole cell extract from monocytes and five micrograms from lymphocytes was resolved by SDS-PAGE and western blotted for Dicer and β-actin. As expected, Western blot analysis revealed that primary lymphocytes express Dicer, but that Dicer levels in primary monocytes were sub-optimal (Fig 1C, compare lanes 1–3 with 4–5). To further examine expression of Dicer in monocytes additional western blotting and quantitative RT-PCR was performed (Fig 1D and 1E). Decreasing amounts of CEM extract were used for western blot (Fig 1D, lanes 1–3) showing detection of Dicer down to 5 micrograms of extract. Western blotting 100 micrograms of U937 or U1 extract failed to detect Dicer (lanes 4 and 5). To confirm these results cell lysates were prepared using a 7 M Urea, 2 M Thiourea buffer (lanes 6 and 7) or through loading 100,000 cells directly onto the gel after lysis in laemlii buffer (lanes 8 and 9). These alternative methods of protein extraction should provide greater solubility of proteins. However, these methods of lysis also yielded very little detectable Dicer, even when 50 micrograms of extract was loaded on the gel. Quantitative RT-PCR indicates that Dicer mRNA is expressed in the CEM and ACH2 cell lines at comparable levels. Interestingly, Dicer mRNA is detectable in U937 and U1 but at 40–60% less than the levels in CEM and ACH2 (Fig 1E). These analyses suggest that monocytes produce a level Dicer that may be sub-optimal for Dicer driven cleavage of RNA, while T-cells express high levels of Dicer.

To address the possibility that monocytes may express Dicer upon differentiation to macrophages, U937 and THP-1 cells were differentiated by treatment with 10, 20 or 100 uM phorbol-myristate acetate (PMA) for 48 hours (Fig. 1F). Differentiation of U937 cells led to the production of low levels of Dicer, however the level of Dicer expression was 2–4 times lower than levels in T-cell lines (compare lanes 2–4 to 9 and 10). This is in keeping with work in the field that has used shRNA, which would require Dicer cleavage, in macrophages [56,57].

These findings are interesting when considered in the context of latent HIV infection, a phenomenon found in CD4+ T-cells, but not in monocytes and macrophages [38,54].

### Dicer binds to the HIV-1 TAR stemloop

Several pieces of information suggest that Dicer may associate with TAR RNA; Dicer is present in HIV-1 target cells, TAR bears resemblance to known Dicer substrates, recent computer modeling and the presence of a double-stranded RNA binding domain in Dicer [28,58]. Association of TAR containing transcripts with Dicer may lead to the production of a TAR derived miRNA

**Figure 2:**
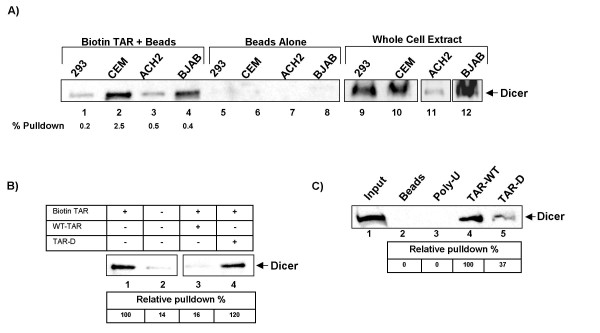
**Cellular Dicer associates with the HIV-1 TAR stem and loop structure**. **A) **Biotin labeled TAR-RNA was incubated with one milligram whole cell extracts from 293, CEM, ACH2 and BJAB (lanes 1–4), pulled down with streptavidin beads, washed thrice with TNE-150, resolved by SDS-PAGE and detected for Dicer by western blot. One milligram cell extract incubated without biotin-TAR was used as a control (lanes 5–8) and 30 microgram whole cell extract from 293 and CEM cells were included in the western as a positive control for Dicer detection (lanes 9 and 10). **B) **Unlabeled wild type (TAR-WT) and mutant TAR (TAR-D) were used in a competition experiment to examine the specificity of Dicer binding to TAR. 293 cell extracts were incubated in the presence (lane 1,3,4) or absence (lane 2) of 1.5 micrograms of biotin-labeled TAR. A 2.4 fold excess of TAR-WT (lane 3) or TAR-D (lane 4) was incubated with two of the samples. Biotin-TAR was pulled down with streptavidin beads and detected for Dicer by western blot as in panel A. Densitometry was performed to determine the relative amount of Dicer present after the pulldown, as compared to the positive control (lane 2). **C) **Pulldown was performed as in panel A, but included a biotin-PolyU and TAR-D in addition to the WT-TAR. Percentages indicate the relative pulldown as compared to TAR-WT.

In order to determine whether the HIV-1 TAR element is recognized by Dicer we performed pull-down assays using a biotin-labeled TAR. Biotin-labeled wild type TAR was used to pull down the Dicer protein from 293, CEM, ACH2 and BJAB cell lines. Dicer is detectable by western blot after pull-down from each of the four cell types tested, but not after incubation with streptavidin beads alone (Fig 2A compare lanes 1–4 with 5–8). Comparison of pull-down to the control lanes for CEM and 293 cells reveals that incubation with biotin-TAR is capable of pulling down 0.3–2.8% of the total cellular Dicer. Western blotting of the biotin labeled TAR pull-downs revealed no increase over background when staining for β-actin, indicating a specific enrichment in TAR associated proteins (data not shown). This shows that Dicer, expressed in CD4+ T-cells and HIV-1 infected T-cells, binds to the HIV-1 TAR structure and implies that the TAR element present in HIV-1 infected or latently infected cells may be processed by Dicer to yield a viral miRNA.

**Figure 3:**
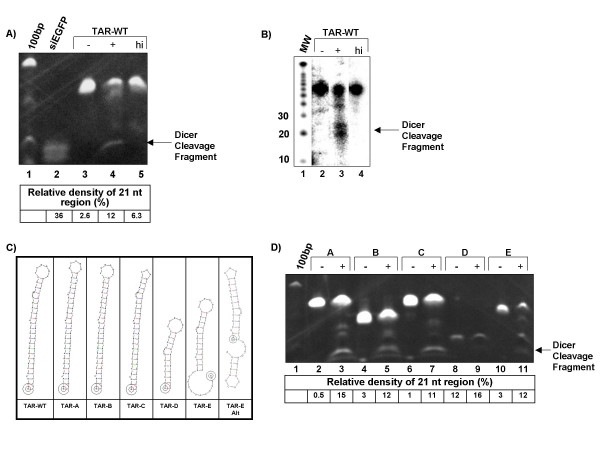
**Dicer cleaves the HIV-1 TAR structure *in vitro***. **A) ***In vitro *transcribed HIV-1 TAR was incubated overnight in the presence of recombinant Dicer or heat-inactived (95° for two minutes) recombinant Dicer, separated on a 6% TBE-UREA gel and stained with ethidium bromide. A 100 bp DNA ladder (lane 1 – band visible at the top of the gel corresponds to 100 bp) and commercially available siRNA against siEGFP (lane 2) were included as size standards. Lane 3 – one microgram *in vitro *transcribed TAR. Lane 4 – one microgram TAR incubated with rDicer. Lane 5 – one microgram TAR incubated with heat inactivated Dicer. Arrow indicates the ~21 nt band generated by Dicer cleavage. Densitometry indicates the signal in the 21 nt region of the gel relative to undigested TAR. **B) **Digest was repeated as in panel A. Products were resolved on a 15% TBE-Urea gel and visualized by autoradiography. MW (lane 1) was exposed for 5 minutes, while the digests (lanes 2–4) were exposed for 2 hours. Numbers to the left indicate the size of the associated RNA marker. **C) **Predicted structures of the TAR mutants used for Dicer cleavage analysis. TAR-A is a compensatory mutant, where the base pairs in the stem have been switched. TAR-B has the bulge (TAR binding site) deleted. TAR-C has a deletion in the terminal loop. TAR-D has a truncated stem. TAR-E has a shortened stem and a long 5' tail, or may fold into a two looped structure. **D) **TAR mutants pictured in panel B were digested with Dicer, separated on a TBE-UREA gel and stained with ethidium bromide. Lane 1 – 100 bp marker. Pictured are mutants TAR elements before and after Dicer treatment; TAR-A (lanes 2 and 3), TAR-B (lanes 4 and 5), TAR-C (lanes 6 and 7) and TAR-D (lanes 8 and 9). Densitometry was performed as in panel A, where the intensity of the 21 nt region of the gel is shown in relation to the undigested mutant.

To ascertain whether or not pulldown of Dicer by TAR is specific to the structure of TAR, and not due merely to the presence of RNA, we performed competition experiments using unlabeled TAR and a TAR mutant. 293 cell extracts were used for pulldowns to test the ability of wild type and mutant TAR to compete with the biotin labeled TAR for Dicer binding (Fig 2B). A TAR mutant, designated TAR-D, was chosen that contains an extensively shortened stem region, which we predicted would prevent Dicer binding and processing (TAR-D sequence is shown in Table 1, top panel, and in Fig 3B). Pull-downs were performed as above, with the addition of two conditions wherein 3.4 micrograms (a 2.4 fold excess) of either wild type TAR or TAR-D (Fig 2B lanes 3 and 4 respectively) was included during the incubation of extract with biotin labeled TAR. Biotin-TAR associated proteins were pulled-down by incubation with streptavidin beads and western blotted for the presence of Dicer. Initial examinations of the Western blot revealed that an unlabeled WT-TAR is capable of competing with the biotin labeled TAR for Dicer binding, while the TAR-D mutant does not. Densitometry was performed to determine the relative amount of Dicer present after each pulldown as compared to the positive control (lane 2). Incubation with unlabeled wild type TAR decreased the amount of Dicer detectable after the pulldown to 16% of the positive control, or just above background (Fig 2B, lane 3). Incubation with TAR-D had no effect on the ability of biotin labeled TAR to pulldown Dicer from whole cell extracts (lane 4).

**Table 1:** Sequence of HIV-1 TAR, TAR mutants, oligonucleotides used to generate RPA probes, and synthetic siRNA

Name	Sequence
TAR-WT	GGUCUCUCUGGUUAGACCAGAUCUGAGCCUGGGAGCUCUCUGGCUAACUAGGGAACC
TAR-A	GCACAGGGAUCAAUCAGGUCUUCUCUCGCUGGGACGAGAGACCGAUUGGUCUCUUGC
TAR-B	GGUCUCUCUGGUUAGACCAGA___GAGCCUGGGAGCUCUCUGGCUAACUAGGGAACC
TAR-C	GGUCUCUCUGGUUAGACCAGAUCUGAGCCU___AGCUCUCUGGCUAACUAGGGAACC
TAR-D	GG______________CCAGAUCUGAGCCUGGGAGCUCUCUGG____________CC
TAR-E	GGUCUCUCUGGUUAGACCAGAUCUGAGCCUGGGAGCUCUCUGGCUA___________
	
T7 Primer	TAATACGACTCACTATAGGGAGA
TAR 5' Template	GGTCTCTCTGGTTAGACCAGATCTGATTTTTTCTCCCTATAGTGAGTCGTATTA
TAR 3' Template	AGCTCTCTGGCTAACTAGGGAACCCACTTTTTTCTCCCTATAGTGAGTCGTATTA
	
siGL2	5' CGUACGCGGAAUACUUCGAUU 3' 3' UUGGAUGCGCCUUAUGAAGCU 5'
siGFP	5' GCGACGUAAACGGCCACAAGUUCUC 3' 3' GCCGCUGCAUUUGCCGGUGUUCAAG 5'
siTAR1	5' UGGGUCUCUCUGGUUAGACCAGUU 3' 3' UUACCCAGAGAGACCAAUCUGGUC-P 5'
siTAR2	5' CUCUCUGGCUAACUAGGGAACCUU 3' 3' UUGAGAGACCGAUUGAUCCCUUGG-P 5'
	
LTR forward	CGAGCTTGCTACAAGGGACT
LTR reverse	GAGATTTTCCACACTGACTAAAAGG
	
Luc forward	TGAACTTCCCGCCGCCGTTGT
Luc reverse	TTACAATTTGAACTTTCCGCC
	
DicerRT F	TTTGTGCAGTTTCAGCTTGA
DicerRT R	CCATGGCCTTTGGAACTTC

To further verify the specificity of Dicer binding to the wild type TAR structure and not the TAR-D mutant the pulldown was repeated using a biotin labeled poly-U (Fig 3C). One milligram of 293 extract was incubated with no RNA (lane 2), a 52 nucleotide biotin-poly-U RNA (lane 3), biotin labeled wild type TAR (lane 4), or a biotin labeled TAR-D mutant (lane 5). The poly-U RNA, which lacks extensive secondary structure, was incapable of binding to Dicer. TAR-D was capable of binding Dicer, but much less efficiently than the wild type (37% to 100%). As TAR-D was incapable of competing with TAR-WT for binding (Fig 2B), we believe that Dicer is not binding directly to this mutant. TAR-D contains a high affinity binding site for TRBP, between the pyrmidine bulge and the terminal loop [45,46,59]. As TRBP is the cellular binding partner of Dicer, this association may allow TAR-D to pulldown Dicer that is associated with TRBP [43,44].

Therefore, these experiments demonstrate, for the first time, that the Dicer protein can bind to the HIV-1 TAR element. Also, the competition experiments show that this association of Dicer and TAR is specific to the TAR sequence and cannot be disrupted by the addition of a mutant that has a portion of the TAR stem deleted.

### Dicer cleaves the HIV-1 TAR stemloop, but not stemloop mutants

To test the ability of the TAR element to be cleaved by Dicer, we incubated *in vitro *transcribed TAR with a recombinant Dicer and used ethidium bromide staining to detect cleavage products on a 6% TBE-Urea polyacrylamide gel. Incubation of WT-TAR with Dicer yielded an RNA fragment of approximately 21 nucleotides, which was not present in samples incubated with a heat inactivated Dicer, indicating that the small fragment was a Dicer generated miRNA (Fig 3A, compare lane 4 to 5). The relative density of the 21 nucleotide fragment in lanes 2 through 5 was determined as compared to background in the control lanes. An increase of approximately 5 fold over background is seen in the lane with the Dicer digested TAR. This level of cleavage was consistent across three separate experiments. Various titrations for the concentration of NaCl and MgCl_2 _in the digestion buffer provided with the recombinant Dicer did not result in any increase in cleavage of TAR RNA (data not shown). This incomplete *in vitro *cleavage, even under optimal conditions, appears to be consistent with other studies that have evaluated the conditions for optimal Dicer activity [60].

In an attempt to provide greater resolution of the Dicer cleavage product, and obtain greater accuracy as to the size of the product, the cleavage of TAR-WT was repeated using a radio-labeled RNA substrate (Fig 3B). After digestion, the resulting bands were separated on a 15% TBE-Urea gel and visualized by autoradiography. This analysis allowed the use of a small RNA marker, and shows that the cleavage product is running at approximately 21 nucleotides. However, the overall resolution of this gel is not as sharp as the ethidium bromide stained poly-acrylamide.

A panel of TAR mutants (Fig 3C), that have previously been used to evaluate the importance of TAR, was also tested for the ability to be cleaved by Dicer [61-64]. The TAR-A mutant, which contains inversions of each base pair in the stem, the TAR-B mutant, which contains no pyrmidine bulge and TAR-C, with mutations in the terminal loop are all processed by Dicer to yield a small RNA product of approximately 21 nucleotides (Fig 3D, lanes 2–7 and 10–11). Densitometry was performed to compare the intensity of the miRNA product band to background. In mutants A, B, and E this product band was measured as an increase over background of 30, 4, and 11-fold respectively. This increase correlates to the one seen in Dicer processing of the wild type TAR RNA. The short TAR-D mutant was not cleaved by Dicer (compare lane 8 to 9). Incubation of TAR-D with Dicer did not produce any detectable 21 nucleotide fragments. Mutations in the sequence of TAR that maintained the overall integrity of the stemloop are still processed by Dicer. Significant shortening of the stem beyond 20 nucleotides, as with TAR-D, alleviates the ability of Dicer to catalyze cleavage of the RNA. Collectively, these results suggest that Dicer requires a stretch of dsRNA of approximately twenty nucleotides to have an effect, a finding that is consistent with other studies on Dicer activity [42,58]. The need for a 20 nucleotide stretch of dsRNA likely explains why TAR-D, which contains a stem of only 11 bases in length, was not cleaved in our assays.

The TAR-E mutant provided an unexpected result. At first we predicted that this mutant would not be cleaved by Dicer due to its shortened size. However, repeated experiments have shown that it is, in fact, cleaved by Dicer. We believe this to be occurring for one of three possible reasons; 1) the double-stranded portion of TAR-E is actually 2 bases longer than that of TAR-D and could perhaps alter Dicer's ability to bind, 2) The long 5' tail may somehow facilitate Dicer binding, 3) TAR-E may actually fold differently than predicted and form a longer hairpin. In support of the third possibility we have predicted what TAR-E would look like when the instability created by unpaired stretches of RNA is considered (Fig 3B, TAR-E Alt). This structure consists of two closely connected stem loops, which now provide the short 3' overhang favored by Dicer and increased the overall double-stranded character of the structure. This alternative structure may facilitate Dicer binding and cleavage.

### HIV-1 positive cell lines produce a TAR derived miRNA

**Figure 4:**
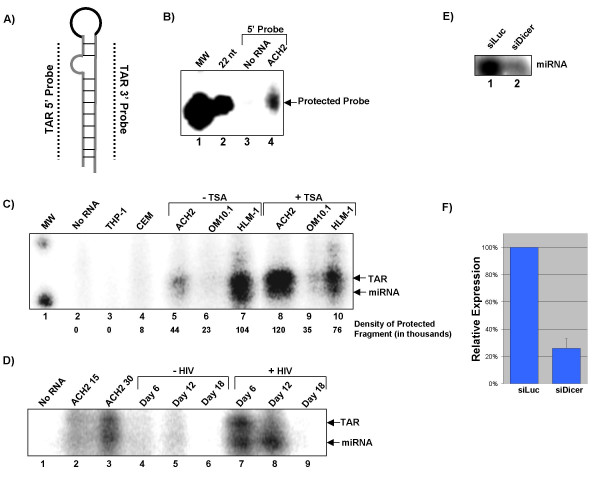
**HIV-1 infected T-cell lines produce a miRNA derived from the TAR element**. **A) **Graphical representation of what region of TAR is detected by the probe the probe used for RPA. TAR 5' probe detects sequence from the 5' area of the stem (~30 bases), while TAR 3' detects the 3' side of the stem. **B) **Thirty micrograms of total RNA from ACH2 was hybridized to a radiolabeled RNA probe specific for 5' (lanes 3 and 4) region of the TAR stem loop and then treated with RNase A and T1. As a control the probes were incubated alone with RNase A and T1 (lanes 3). Molecular weight marker corresponding to 20 nucleotides is shown (lane 1) as well as a 22 nucleotide standard (lane 2). **C) **Thirty micrograms of total RNA from THP-1, CEM, OM10.1, (lanes 3 and 4) ACH2, OM10.1 and HLM-1 (lanes 5–7) and from OM10.1, ACH2 and HLM-1 stimulated with TSA for 24 hours (lanes 8–10) were hybridized to a radiolabeled TAR 5' probe and then treated with RNase A and T1. As a control probe was incubated alone with RNase A and T1 (lane 2). Molecular weight markers corresponding to 20 and 30 nucleotides are shown (lane 1). Arrows indicate the probe protected by TAR at 27 nucleotides and probe protected by a TAR miRNA at approximately 22. **D) **T-cell blasts were cultured from PBMCs using IL-2 and PHA for 48 hours. T-cells were infected with HIV-1 (LAV) and cultured in RPMI with IL-2 for 6, 12 and 18 days. RNA from uninfected cells at day 6, 12 and 18 (8.3, 3.7 and 16.8 micrograms respectively – lanes 4–6) and infected cells at 6,12 and 18 (9.2, 16.5 and 22.9 micrograms – lanes 7–9) was probed for presence of TAR derived miRNA using the 5'TAR probe. A sample containing no RNA was included as an additional negative control as were 15 and 30 micrograms of ACH2 RNA (lanes 1–3). **E) **HLM-1 cells were transfected with siLuc or siDicer for 72 hours. RNA extract was used to perform RPA for HIV-1 miRNA. **F) **Expression of HIV-1 TAR miRNA was determined by densitometry and normalized to 5S ribosomal RNA.

In order to ascertain whether or not HIV-1 infected cells produce a TAR derived miRNA, we utilized RPA to detect small RNA fragments corresponding to TAR sequence. Probes were designed which were complementary to the entire length of the 5' or 3' portion of the TAR stem loop and would detect the generation of ~21 nt RNAs from any position (Table 1 and Fig 4A).

Initially we sought to detect the presence of a TAR derived miRNA in the ACH2 cell line. ACH2 are chronically infected CD4+ T-cells, which produce virus at low levels but can be stimulated to high levels of virus production with TNF-α, sodium butyrate or trichostatin-A. Thirty micrograms of total RNA from ACH2 was probed for TAR derived miRNA by RPA using the TAR 5' probe or 3' probe (Fig 4B). RNase protection assay revealed the presence of an HIV-1 derived miRNA in ACH2 corresponding to the 5' (lane 4) but not the 3' portion of TAR (Data not shown). Detection of only one strand of the duplex is consistent with the current understanding of miRNA biogenesis. After the cleavage of double stranded RNA by Dicer one strand of the 21 nucleotide duplex is guided by Dicer and TRBP into the RISC complex to become the guide strand. The other strand is degraded, likely through the action of RISC [65-68]. The presence of a TAR derived miRNA in ACH2 cells was also supported by northern blot analysis (data not shown).

We sought to assay the presence of TAR derived miRNA in several cell types. As we hypothesize that a TAR generated miRNA may be involved in viral latency, we sought to test cell lines that would serve as a cell culture model of latency. We chose cell lines that are infected with HIV-1, produce a low or undetectable level of virus, and can be stimulated to produce high levels of infectious virus. We selected ACH2 again as a control. For cells of myeloid lineage, we selected the chronically infected OM10.1 pro-myelocytic cells; the only cells of the monocyte lineage in which we detected Dicer expression. These cells are latently infected and can be stimulated, much like ACH2, to produce high levels of full-length transcript and infectious virus. Finally, we chose HLM-1, a HeLa derivative that is stably transfected with an HIV-1 provirus and is wildtype for all ORFs except Tat. Much like the OM10.1 and ACH2, the HLM-1 cells produce no detectable virus but can be stimulated with TNF-α, sodium butyrate, trichostatin-A or Tat to produce infectious virus.

Thirty micrograms of total RNA from either unstimulated HLM-1, ACH2, OM10.1 or cells stimulated for 24 hours with 450 nM Trichostatin-A was probed for TAR derived miRNA by RPA using the TAR 5' probe. RNase protection assay revealed the presence of an HIV-1 derived miRNA corresponding to the 5' portion of TAR in ACH2, OM10.1 and HLM-1 cells (Fig 4C, lanes 5–7). Densitometry was performed to determine the relative levels of the TAR miRNA in each cell type. Stimulation of these cells with trichostatin-A, which induces high levels of HIV-1 expression, resulted in an increase in expression of this miRNA in ACH2 and OM10.1 by 2.8 and 1.5 fold respectively (Fig 4A, compare lanes 5 and 6 to 8 and 9). Additionally, this second analysis allowed us to resolve two bands in the RPA. The top band migrates at approximately 27 nucleotides and represents probe protected by full-length TAR, while the lower band migrates at 22 nucleotides and represents probe protected by hybridization to a TAR-derived miRNA. Knowing that our RPA was capable of detecting full length TAR and the miRNA, we wondered why our probing of ACH2 with TAR3' did not reveal a full length TAR product (Fig 4B). We believe this lack of detectable product is due to a previously characterized mutation within the TAR element of the ACH2 cell line [69]. This mutation falls within the area probed by TAR3' and would lead to loss of protection and subsequent degradation of the probe by RNase.

To examine the presence of TAR derived miRNA in *de novo *infection of T-cell blasts from a healthy donor, PBMCs were infected with HIV-1 and grown in culture for 18 days. RNA was harvested from infected and uninfected cells at days 6, 12 and 18. RNA extracts from these time points were analyzed by RPA for the presence of the TAR derived miRNA detected in panels B and C (Fig. 4D). RPA revealed the presence of a 22 nucleotide miRNA in the day 6 and 12 infection, but not in the uninfected cells (compare labes 4 and 5 to 7 and 8). Interestingly, on day 6 the ratio of TAR to miRNA is about equal, whereas on day 12 the majority species is the miRNA. This suggests that production of the TAR derived miRNA may continue, even as viral transcription is reaching its peak and ending. We were unable to detect either TAR or TAR miRNA on day 18, likely due to the limited life span of infected T-cell blasts in culture.

To confirm that the 22 nucleotide RNA product is indeed a miRNA yielded by Dicer acting upon the TAR element we knocked-down the expression of the Dicer protein in HLM-1. HLM-1 were transfected with siDicer (Dharmacon) or a control siRNA against Luciferase (siLuc) and cultured for 72 hours. RNA was prepared from HLM-1 at 72 hours post transfection and used for RPA analysis of miRNA expression (Fig 4E). Expression of TAR derived miRNA was measured by densitometry and normalized to signal from the 5S ribosomal RNA (Fig 4F). Analysis revealed that knockdown of Dicer expression for 72 hours reduced the level of TAR miRNA expression by approximately 75%. This indicates that the 22 nucleotide band detected by RPA is indeed a Dicer generated miRNA.

These RPA analyses present evidence that the HIV-1 TAR structure is processed into miRNA *in vivo*. This TAR derived miRNA can be detected in latently infected cell lines and stimulation of these cells line increases miRNA expression by approximately two-fold. Additionally, this miRNA can be detected in acute infection of primary T-cells.

### siRNA directed against the HIV-1 LTR down regulates LTR driven gene expression

A miRNA generated from one side of the HIV TAR stemloop, such as the small RNA detected by the TAR 5' probe in our RPA assay, will possess significant similarity to the complementary strand of the TAR element. Production of this viral miRNA may down regulate viral gene expression by targeting the RISC or RITS complexes to viral RNA transcripts that contain the TAR element.

The target region for our proposed miRNA lies within the secondary structure of TAR. As previous reports have indicated that extensive secondary structure may prevent access of RISC to the mRNA and render the sequence less susceptible to RNAi mediated down-regulation [70,71], we sought to determine if HIV-1 TAR is susceptible to regulation by RNAi.

We tested the ability of TAR derived miRNA to downregulate viral gene expression through the use of a plasmid containing the luciferase gene under control of the HIV-1 LTR. Transcription initiated by the viral LTR will produce a luciferase mRNA with the TAR structure at the 5' end. Transfection of *in vitro *transcribed TAR RNA into a Dicer producing cell should allow processing of the TAR stemloop by cellular Dicer and generate the same miRNA detected in our Dicer cleavage assays. As such, we transfected 293 cells in a 96-well plate with 100 nanograms of pLTR-Luc, 100 nanograms of pRL-CMV and 25 nanograms of RNA. Cells were transfected with either *in vitro *transcribed wild type TAR (TAR-WT), TAR mutant D or A (TAR-D or TAR-A). TAR-D was selected as this mutant did not yield a miRNA fragment after incubation with Dicer in our Dicer cleavage assay and was incapable of competing with wild type TAR for Dicer binding. TAR-A was selected as the large sequence variations in the stemloop would lead to production of a miRNA incapable of targeting an RNAi effector complex to native TAR sequence. The firefly Luciferase specific siGL2 was included as a control and the amount of siRNA in each well was adjusted to 25 nanograms with siGFP, an siRNA against enhanced green fluorescent protein, which serves as a negative control in these experiments (siRNA sequences are depicted in Table 1). Transfection efficiency was normalized by transfecting a renilla luciferase under control of the CMV promoter along with the LTR constructs. Data presented represents the ratio of LTR driven expression to CMV driven expression, allowing specific determination of the effect of various RNAs on the LTR constructs.

**Figure 5:**
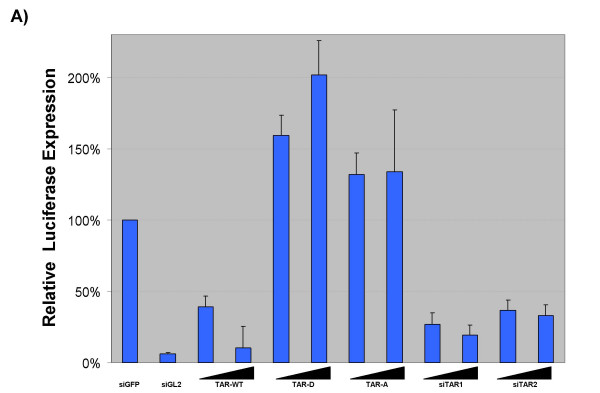
**siRNA directed against the HIV-1 TAR structure can potentially downregulate LTR-driven viral expression**. 293 cells were transfected with 100 nanograms of pLTR-Luc, 100 nanograms of pRL-CMV and 25 nanograms of RNA and assayed 48 hours later for firefly and renilla luciferase expression. Cells were transfected with 25 nanograms siGFP, 25 nanograms siGL2, 5 and 25 nanograms of TAR-WT, TAR-D, TAR-A, siTAR1 or siTAR2. RNA levels were normalized to 25 nanograms using siGFP.

Wild type TAR RNA was capable of suppressing Luciferase expression as compared to siEGFP (Fig 5). Luciferase specific siRNA (siGL2) induced a ~95% decrease in luciferase activity as compared to control. TAR-WT suppressed luciferase expression by ~60% at the 5 nanogram level and 80% at the 25 nanogram level. Transfection of the TAR mutants TAR-D and TAR-A had no significant effect on luciferase activity. These results indicate that delivery of wild type TAR RNA into cells decreases expression of a protein driven by the HIV-1 LTR. This effect is likely due to processing of TAR element by Dicer and incorporation of the resulting miRNA into the RISC or RITS complex.

To further clarify whether TAR is available for regulation by RNAi, we tested the ability of TAR specific siRNAs (obtained from Dharmacon) to block LTR driven transcription of luciferase. These TAR specific siRNAs have a 5' phosphate on the antisense strand, which should enhance incorporation of the antisense strand into the RISC complex. This directs siTAR1 to specifically target the first half (5') of the TAR element and siTAR2 to target the second (3') half (indicated by underline in Table 1, top panel).

Delivery of siTAR1 or siTAR2 into 293 cells led to a suppression of LTR-driven luciferase activity. Transfection of 5 nanograms of siRNA resulted in a 75% loss of luciferase activity, while transfection of 25 nanograms of siRNA resulted in only a slight improvement in suppression of the lower concentration. The 75% suppression at 5 nanograms is greater than the 60% suppression triggered by transfection of TAR-WT RNA, suggesting that the siTAR1 is slightly more efficient at mediating suppression than the *in vitro *transcribed TAR.

It should be noted that the luciferase specific siRNA is targeted against the middle of the luciferase mRNA, an area that likely does not contain extensive secondary structure like the TAR element. The absence of secondary structure in the target region for siGL2 may explain the fact that TAR-WT RNA as well as siTAR1 and siTAR2 are incapable of suppressing luciferase expression to the same level as siGL2. This data shows that RNAi can be directed against the TAR element, in spite of its extensive secondary structure. The HIV-1 TAR derived miRNA detected in the RPA is derived from the first half of the TAR structure and is complementary to the later half of TAR, much like the siTAR2. These findings suggest that the TAR derived viral miRNA detected in ACH2, OM10.1 and HLM-1 cells could potentially be down regulating viral replication and contributing to the low level of viral gene expression seen in these cells. Although the induction of virus in these cell types with TSA altered the production of viral miRNA differently in each cell type, when considering that transactivation by Tat increases viral transcription several-hundred fold; it is likely that the increase in overall transcription overcomes any blockage due to RNAi [72-74].

### TAR derived miRNA causes HDAC-1 to associate with the viral LTR

Recent studies have shown that siRNA targeted to the NF-κB sites within the viral LTR leads to transcriptional silencing through chromatin remodeling [75]. Additionally it has been suggested that a TAR derived miRNA may have similar effects [76]. To test the ability of a TAR derived miRNA to direct chromatin remodeling at the viral LTR we used chromatin immunoprecipitation to examine the recruitment of HDAC-1, a histone deacetylase shown to be involved in silencing of HIV, to the LTR of the pLTR-luc plasmid [77].

**Figure 6:**
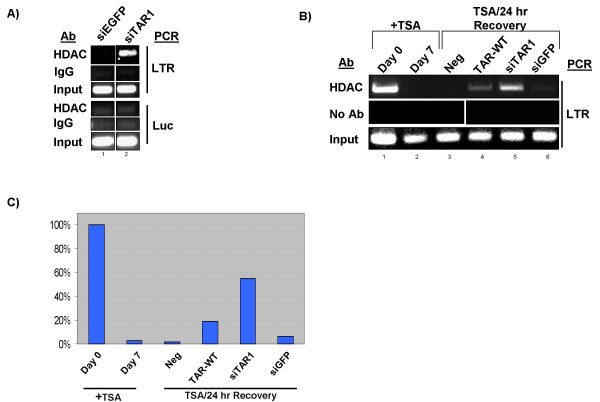
**HDAC-1 is recruited to the HIV-1 LTR by TAR derived miRNA**. 293 cells were transfected with pLTR-Luc and either siGFP or siTAR1. After 48 hours cells were crosslinked with formaldehyde, lysed, sonicated and immunoprecipitated for HDAC-1 or a IgG antibody. **A) **PCR on DNA from HDAC-1 ChIP, control IgG ChIP and input was performed for either LTR or luciferase (Luc) region. **B) **TZMbl cells were cultured in the presence of 100 nM TSA for 0 or 7 days (lanes 1 and 2), and then allowed to recover in the absence of TSA withouth (lane 3), with either TAR-WT or siTAR1 transfection (lanes 4 and 5), or transfection with siGFP (lane 6). PCR for LTR was performed on HDAC-1 ChIP DNA (top panel), no antibody control (middle panel) or input DNA (bottom panel). **C) **Densitometry was performed on the gel shown in panel B. Background signal in the no antibody PCR was subtracted from the HDAC-1 ChIP and results are shown as a percentage of HDAC-1 association with the LTR before TSA treatment.

293 cells were transfected with pLTR-Luc and either siEGFP or TAR-WT RNA. Following crosslinking, lysis and shearing of DNA by sonication, lysates were immunoprecipitated with an antibody against HDAC-1. Association of HDAC-1 with either LTR or luciferase DNA was determined by PCR (Fig. 6A). Primers were designed to detect the region surrounding transcription start (-119 to +175) and an area ~2000 bases downstream of the transcription site in the luciferase ORF. ChIPed DNA was diluted 1:20 and subjected to PCR for LTR sequence (panel A top panel). Comparison of siTAR-1 transfection to siEGFP control suggests that siTAR-1 RNA is capable of driving HDAC recruitment at the LTR. Control analyses for unrelated antibody and input were also performed (second and third panels). To examine the level of HDAC-1 recruitment downstream of the LTR PCR was performed using luciferase specific primers (Fig 6A bottom two panels). This analysis showed that no recruitment of HDAC-1 could be detected 2000 bases downstream from the miRNA target site. This suggests specific recruitment of HDAC-1 to the promoter region complementary to the TAR miRNA or synthetic siRNA.

To further verify the involvement of TAR derived miRNA in chromatin remodeling we sought to test its effects on an integrated HIV-1 LTR. For this we utilized TZMbl cells, a HeLa derivative that carries two integrated copies of the HIV-1 LTR; one driving luciferase and the other driving beta-galactosidase. These cells express low levels of luciferase in the absence of stimulation, suggesting that the LTR is already silenced. Previous work in the field has shown that chronic treatment of HeLa cells with the HDAC inhibitor TSA abolishes the heterochromatic state [78]. Indeed, a similar approach has been used to study chromatin in yeast and elucidate the involvement of RNAi [79,80]. As such we treated the cells for seven days with the HDAC inhibitor TSA. This treatment induced detectable levels of luciferase gene expression, which began to return to pre-treatment levels after the removal of TSA (data not shown). This suggested to us that after the removal of TSA the DNA was returning to the heterochromatic state and silencing gene expression.

We employed this system to analyze the effect of TAR and siTAR1 on HDAC-1 recruitment to the integrated HIV-1 LTR. TZMbl cells were treated with 100 nM TSA for 7 days. On day 6 the cells were transfected with either TAR-WT, siTAR1 or and siGFP control. On day 7 TSA was removed, and on Day 8 cells were ChIPed for HDAC-1 or no antibody control (Fig 6B). Before treatment HDAC-1 was associated with the LTR and this association was lost upon treatment with TSA (compare lanes 1 and 2, top panel). Transfection of the cells with TAR-WT or siTAR1 RNA led to an increase in the recovery after 24 hours as compared to the control (compare lane 3 to 4 and 5, top panel). Control transfection with siGFP resulted in a level of HDAC-1 recruitment similar to the untransfected cells (compare lane 6 to 3). PCR using no-antibody ChIP and input DNA showed comparable detection of LTR DNA in all lanes (middle and bottom panels). Densitometry was performed to analyze the relative levels of HDAC-1 recruitment to the integrated LTR (Fig 6C). Background signal in the no antibody lanes was subtracted from the HDAC-1 signal and displayed as a percentage of HDAC-1 on the LTR at day 0. This analysis revealed a 97% drop in HDAC-1 association with the LTR after TSA treatment (100% to 2.9%). Twenty four hour recovery, or transfection with siGFP, resulted in a slight reconstitution of HDAC-1 (1.6% and 6.0% respectively), while transfection of cells with TAR or siTAR1 greatly accelerated the recruitment of HDAC-1 to the LTR (18.8% and 54.8% respectively). Analysis of HDAC-1 recruitment to an integrated LTR verifies that heterochromatin formation at the HIV-1 LTR is driven by RNA interference mediated by the TAR derived miRNA. This effect is specific to the transfection of an siRNA homologous to TAR sequence or to the TAR hairpin, and is not seen with the control transfection.

The de-acetylation of histones by members of the HDAC family is a key step in the formation of heterochromatin. Modifications of the histone tails leads to condensation of the chromatin structure and silencing of transcription at the affected locus [81]. RNAi has been shown to be capable of silencing transcription through the RITS complex by recruiting chromatin remodeling factors to a complimentary sequence of DNA [13,14,16]. The recruitment of HDAC-1 to the HIV-1 LTR in presence of either TAR specific siRNA or TAR RNA demonstrates that HIV-1 derived miRNA is capable of silencing gene expression through transcriptional gene silencing. The lack of HDAC-1 in the luciferase ORF, approximately 2000 bases downstream, demonstrates the specificity of this recruitment to the complementary sequence in the LTR.

## Discussion

RNAi is a recently discovered regulatory mechanism, the specificity of which is guided by small RNA molecules that direct a silencing complex to a complementary sequence of mRNA [1,3,82]. Based upon the precursor from which these RNAs are derived they are termed siRNA or miRNA. siRNA is generated from a long sequence of double stranded RNA that is processed by a cellular ribonuclease III enzyme called Dicer. miRNA is generated from endogenously expressed RNA that folds into a hairpin structure. This hairpin is cleaved in the nucleus by an enzyme called Drosha, exported to the cytoplasm and processed by Dicer [2,4,7,9,83]. As the final processing step in the generation of siRNA and miRNA is catalyzed by Dicer the final products cannot be differentiated. Each is a short double stranded segment of RNA of approximately 21 nucleotides. Of this 21 nucleotide duplex, one strand is incorporated into an effecter complex and targets that complex to a complementary sequence of RNA. RNAi may act post-transcriptionally, by cleaving mRNA or repressing translation, or at the transcriptional level by initiating chromain remodeling and silencing of Pol II mediated gene transcription [1,2,16,82,84].

Although extensive attention has been given to the therapeutic applications of RNAi in the treatment of HIV, relatively little work has been done to examine whether the virus itself may generate miRNA or otherwise subvert the RNAi mechanism for its own benefit [56,57,70,75,85-92]. The sequence specificity of RNAi provides us with a powerful tool for examination of gene function and the potential to combat human diseases, but it should be noted that it represents a natural pathway that has developed as a means to regulate gene expression, protect the integrity of the genome and possibly to defend against viral pathogens [17,18,82,93]. Any virus will have evolved in the context of RNAi and may be subject to suppression by small RNAs. Indeed, it has become apparent that most plant viruses and many mammallian viruses encode a suppressor of RNAi to help counter this potentially detrimental mechanism [94-98]. Recent work has also suggested that many viruses produce their own miRNA [25,26,99,100]. An interesting paper by Triboulet *et al *examines the role of Dicer and Drosha HIV-1 infection [91]. The authors examine the interplay between HIV and the cellular miRNA pathway by knocking out Dicer and examining the effects on viral expression. In the absence of Dicer the virus actually replicates faster. Although Triboulet and colleagues attribute this to alteration in expression of cellular miRNA that have an antiviral effect, they do not rule out the possibility that Dicer is acting upon the viral RNA itself. In this study we provide evidence that that TAR element found at the 5' end of HIV-1 RNA transcripts is processed by Dicer to yield a viral miRNA. We provide further evidence that this miRNA may be supressing virus replication and propose that it may be involved in maintaining viral latency.

First, we analyzed the expression of Dicer in cell types relevant to HIV-1 infection. Western blots revealed that Dicer is expressed in CD4+ T-cells, the primary target of HIV infection, as well as in HIV-1 infected T-cells and primary lymphocytes. Interestingly, monocytic cell lines and primary monocytes isolated from healthy donors expressed sub-optimal amounts of Dicer and macrophages produced much lower levels of Dicer than did T-cells. We believe this assay to be the first demonstration that Dicer, a vital component of the RNAi pathway, is sub-optimaly expressed in monocytes. This finding is intriguing; as we started this examination with the hypothesis that RNAi may be involved in the maintenance of latency in HIV-1 infected CD4+ T-cells. HIV viral latency is a phenomenon unique to T-cells and has not been reported in cells of the monocyte lineage. That monocytes and macrophages express low levels of Dicer lends further credence to this theory. The discovery that macrophages express low levels of Dicer likely has important implications for treatment of HIV-1 with RNAi and our understanding of the interaction of this virus with the RNAi pathway. Indeed, at least two papers have demonstrated that shRNA, which requires cleavage by Dicer, is functional in macrophages [56,57]. This suggests that the level of Dicer expression observed after differentiation is likely sufficient to participate in an active RNAi pathway. Further examination of Dicer expression and activity in monocytes and macrophages will likely improve our understanding of the overall regulation of RNAi.

We showed that Dicer, the protein implicated in creation of miRNA, is capable of binding the HIV-1 TAR RNA element. Cellular miRNA are derived from RNA transcripts, called primary miRNA (pri-miRNA) that fold into long stem and loop structures. Drosha processes these structures into short hairpins of approximately 50–60 nucleotides in length called pre-miRNA. Pre-miRNA is exported to the cytoplasm through the action of Exportin-5 and processed by Dicer into the mature miRNA. The TAR element is an RNA structure that bears significant similarity to pre-miRNA. As such, we reasoned that the excess of TAR available in the form of short viral transcripts may be available for Dicer processing. Using biotin-labeled TAR we were able to pull down Dicer from cell extracts, showing that the TAR structure is indeed recognized by Dicer. Continuing this line of reasoning we tested the ability of recombinant Dicer to process TAR RNA into miRNA. *In vitro *transcribed TAR, when incubated with rDicer yields small RNA of the correct size to be miRNA. Incubation of a battery of mutant TAR structures with Dicer revealed additional information regarding specificity. Mutations in the sequence of the stem, the bulge or the terminal loop did not abrogate Dicer's ability to cleave the TAR RNA. However, shortening of the stem to approximately 11 nucleotides did prevent Dicer cleavage. These findings are in agreement with previous studies on the activity of Dicer [42,58] and provide evidence that mutations in the TAR sequence are unlikely to help HIV avoid the generation of miRNA from the TAR element.

In order to confirm our *in vitro *findings that TAR can be processed to produce a viral miRNA, we used RNase protection to probe HIV-1 infected cells lines for the presence of TAR derived miRNA. RPA revealed that ACH2, an infected T-cell line, OM10.1, an infected pro-myelocytic cell line, HLM-1 and primary T-cell blasts infected *de novo *with HIV-1 expressed an HIV-1 miRNA derived from the 5' portion of the TAR element (Fig 4). Upon stimulation of ACH2 and OM10.1 with TSA we noted an increase in the expression of the viral miRNA. This increase in miRNA expression during activated transcription may argue against a role for RNAi in latency. However, during transactivation the production of viral RNA increases approximately 500-fold, while the level of miRNA expression increases only 2 fold upon stimulation with TSA [72-74]. We believe that the many fold increase in transcription may easily overcome the slight increase in miRNA abundance.

The study of HIV-1 LTR driven transcription has lead to the discovery that short viral transcripts can be detected in cells containing the HIV-1 LTR. Several groups have shown convincing evidence for the presence of short transcripts isolated from primary peripheral blood mononuclear cells [39-41]. These groups isolated resting T-cells from HIV-1 infected patients and examined the length of viral RNA in these cells. Analysis of these samples through RT-PCR indicated the presence of short (somewhere between 50–100 nucleotides) viral transcripts. Work by Jeang and colleagues has shown that production of short transcripts is not due to transcriptional pausing and that the presence of SV40 T antigen may increase the detectable levels of short transcripts in cell lines[101,102]. We propose that the TAR derived miRNA detected by our RPA is a result of Dicer processing of short TAR-containing RNA, as these hairpins represent structures similar to cellular pre-miRNA and would be present in the absence of activated transcription. However, our analysis cannot rule out the possibility that longer viral RNA containing TAR and other viral ORFs may contribute to the production of miRNA after transactivation.

Lastly, we show evidence that a TAR derived miRNA is able to inhibit LTR-driven gene expression, likely through homology to the TAR element found in LTR-driven transcripts. Using a luciferase gene driven by the viral LTR we were able to show that delivery of TAR RNA, but not a TAR mutant into 293 cells was able to suppress luciferase expression (Fig. 5). We show that commercially synthesized siRNA based on TAR sequence are capable of inducing similar effects. Furthermore, we show specific recruitment of HDAC-1 to the LTR by this TAR derived miRNA, suggesting a role for this miRNA in transcriptional silencing of HIV.

When considering the RNAi pathway in the context of viral infection there are four possible outcomes to consider: 1) miRNA generated by the cell acts to regulate cellular genes in response to infection, 2) miRNA generated by the cell acts to suppress viral genes, 3) viral RNA is processed into miRNA/siRNA and suppress viral gene expression, and 4) viral RNA is processed into miRNA/siRNA that suppress cellular gene expression. The role of RNAi in cellular gene control is an accepted mechanism, and perhaps the most well studied aspect of the field [1,3,82]. It is possible that viral infection causes changes in the expression of endogenous miRNA, but this possibility has only recently begun to be explored. Three recent papers discuss the possibility that HIV alters expression of host cell miRNA and show preliminary evidence that this may be the case [91,103,104]. It has been reported that cellular miRNA may have significant homology to certain viruses and that these miRNA serve to protect the cell from specific pathogens [95]. Interestingly, one group has predicted that several cellular miRNA posses homology to the HIV-1 Nef, Vif, Vpr and Vpu genes [50]. Although this prediction has not yet been validated experimentally it does suggest a role for cellular miRNA in regulating HIV-1 gene expression. It has also been demonstrated that siRNA or miRNA generated from viral sequence may be important in limiting viral replication [17,82,93]. Furthermore, the discovery that many viruses encode a suppressor of RNAi suggests that viruses may be under significant pressure from the RNAi mechanism [94,96,105]. Lastly, it should be noted that ongoing work in the field has revealed the presence of viral miRNA expressed by several other viruses [24-26,99].

It is worth noting that a paper from the Tuschl lab, which showed evidence of miRNA in members of herpesvirus family, tested an HIV-1 infected cell line for the presence of miRNA and found none [24]. This paper used a high-throughput method to examine RNA extracts for the presence of short sequences with similarity to known viral sequence. Although this is a powerful technique for screening for unknown miRNA it is likely that this method, which relies on RNA adaptor ligation and subsequent sequencing, may be less sensitive than an RNase protection assay and incapable of detecting low abundance miRNA. We believe our analysis, which used three infected cell lines as well as infection of primary cells, provides conclusive evidence for the existence of an HIV-1 derived miRNA.

## Conclusion

**Figure 7:**
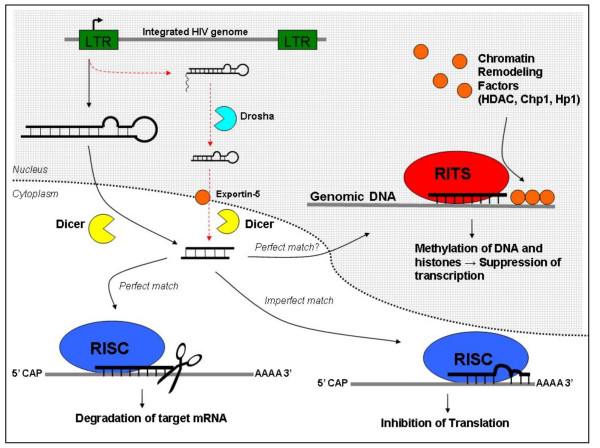
**Model for the generation and action of HIV-1 TAR derived miRNA**. Basal transcription of the HIV-1 LTR produces short RNA transcripts containing the TAR element. These RNA elements are directed to the cytoplasm where they are processed by Dicer to yield a viral miRNA. This viral miRNA associates with the RISC or RITS complex and through homology to sequence contained in the viral RNA, as well as the proviral genome, may direct silencing of viral gene expression. Alternatively, the viral miRNA may contain sufficient homology to mediate silencing of cellular genes. Our work identifies a role for Dicer in the generation of an HIV-1 miRNA, but we cannot rule out the involvement of Drosha *in vivo*. An alternate pathway for the generation of this miRNA is indicated by the dashed arrows. It should be noted that the two pathways may generate miRNA with slightly different sequence, as cleavage by the Drosha enzyme would eliminate some sequence from the base of the stem.

Our work has demonstrated the existence of an HIV-1 miRNA derived from the TAR RNA sequence. We propose that short RNA transcripts of approximately 50 to 100 nucleotides in length are processed by Dicer to yield a viral miRNA. This miRNA may function to suppress viral transcription, a process we propose to be involved in latency, or may even alter the expression of cellular genes through transcriptional or post-transcriptional gene silencing (Fig. 7). The continued and even increased production of viral miRNA after TSA stimulation of latent cells opens the possibility that this miRNA may also play a role in altering cellular gene expression during activated transcription. It should be noted that although we tested the ability of Dicer to act directly on the TAR element, we cannot rule out the *in vivo *involvement of Drosha in processing the TAR RNA prior to its export to the cytoplasm and processing by Dicer. However, regardless of the specific pathway for the production of this miRNA we show *in vitro *evidence for its creation and *in vivo *detection of a TAR derived miRNA through RPA. Furthermore, we have shown the ability of this miRNA to down regulate expression of viral genes. This finding, coupled with the detection of HIV-1 miRNA in un-stimulated HIV-1 infected cells suggests a possible role for this miRNA in maintaining latency. Further work is in progress to elucidate the expression patterns of this miRNA and further investigate the ability of this TAR derived miRNA to influence both viral and cellular gene expression.

## Methods

### Cell Culture, transfections and infections

T-cell lines (CEM, Jurkat, Molt4, Hut78, H9), HIV-1 infected T-cell lines (ACH2, J1.1), monocytes (THP-1), promonocytes (U-937), HIV-1 infected pro-monocytic cells (U1), infected pro-myelocytes (OM10.1) and human B-cell lymphoma cell line (BJAB) were grown in RPMI (Quality Biologicals) supplemented with 10% FBS, 2 mM L-glutamine and penicillin/streptomycin. 293 human fibroblasts, HeLa human cervical carcinoma cells and HeLa cells stably transfected with a Tat deficient HIV-1 (HLM-1) were cultured in DMEM (Quality Biologicals) supplemented with 10% FBS, 2 mM L-glutamine, penicillin/streptomycin and, in the case of HLM-1, G418.

For transfections, 293 cells were seeded in a 96 well culture plate at 15,000 cells/well. The following day the cells were transfected with 100 nanograms luciferase reporter plasmids and with either siGL2, siTAR1, siTAR2, TAR-WT, TAR-D, TAR-A or siEGFP (Dharmacon) using Metafectene (Biontex) lipid reagent (Table 1). Total amount of siRNA was held constant using siGFP. The underlined areas of TAR sequence shown in Table 1 are the areas targeted by siTAR1 and siTAR2.

For infections HIV-1 strain LAV was grown from OM10.1 infected promyelocytes by treatment of the cells with TNF-α. Virus containing supernatant was harvest, filtered to remove cells and concentrated by ultra-centrifugation. PBMCs were cultured in RPMI supplemented with IL-2 and PHA for 48 hours prior to infection. After infection cells were cultured in RPMI with IL-2.

### Isolation of PBMCs

Peripheral blood mononuclear cells were isolated from healthy donor blood according to standard procedures. In brief, 10 ml of blood was layered over 5 ml of ficoll and spun at 1000 × G for 30 minutes with no braking. The white cell layer was removed and washed twice with PBS. PBMCs were plated at ~5 × 10^6 ^cells/well in a 6-well culture plate in DMEM with no FBS. Cells were incubated at 37°C and 5% CO_2 _for one hour to allow adherence. Lymphocyte fraction, including CD4+ T-cells, CD8+ T-cells and B-cells was aspirated from the adhered monocytes. Monocytes were washed once with PBS and scraped from the plate. Lymphocytes and monocytes were then washed once with PBS and lysed to obtain whole cell extracts.

### *In vitro *transcription of TAR and TAR mutants

T7 expression plasmids for TAR and TAR mutants were obtained from the laboratory of Dr. Ajit Kumar (GWUMC) and have been previously described [106]. In the TAR-A compensatory mutant the basepairs have been switched (G-C becomes C-G) while the non-paired nucleotides were unchanged. In TAR-B the pyrmidine bulge was deleted (UCU at position 22). TAR-C contains a deletion in the loop (GGG at position 31). TAR-D contains mutations which shorten the stem region (Fig 3 and Table 1). For *in vitro *transcription reactions 1.5 micrograms of each plasmid was linearized with HindIII (New England Biolabs), ethanol precipitated and used for *in vitro *transcription via the MegaScript High Yield Transcription Kit (Ambion). After transcription TAR RNA was purified on a 2% agarose gel, eluted from the gel with 0.5 M NaAcetate, 1 mM EDTA, 0.2% SDS, and ethanol precipitated before re-suspension in DEPC treated water.

### Biotin-TAR pulldown

One milligram of whole-cell extract from 293, CEM, ACH2 and BJAB was incubated with 1.5 microgram biotin-TAR (a gift of Dr. Sergei Nekhai, Howard University, Washington, DC) and RNase inhibitor at 4°C for two hours. For competition assays, 3.4 micrograms (2.4 fold excess) of wildtype or mutant TAR was added to the mixture. TAR bound proteins were pulled down by incubation with streptavidin-sepharose beads (Amersham) at a 30% slurry for 1.5 hours at 4°C. Unbound proteins were washed three times with TNE-150 +0.1% NP-40. Bound proteins were denatured from the beads and TAR RNA by addition of Laemli buffer and heating to 95°C for two minutes. Proteins were resolved by SDS-PAGE and detected for Dicer by western blot.

### Western blot

Cell extracts and TAR bound protein fractions were resolved by SDS-PAGE on a 4–20% tris-glycine gel (Invitrogen). Proteins were transferred to Immobilon membranes (Millipore) at 200 mA for 2 hours. Membranes were blocked with PBS 0.1% Tween-20 + 5% BSA. Primary antibody against either Dicer (AbCam, AB14601) or Actin (SantaCruz, SC-1615) was incubated with the membrane in PBS +0.1% Tween-20 at 0.5 ug/ml overnight at 4°C. Membranes were washed three times with PBS +0.1% Tween-20 and incubated with HRP-conjugated secondary antibody for one hour. Presence of secondary antibody was detected by SuperSignal West Dura Extended Duration Substrate (Pierce). Luminescence was visualized on a Kodak 1D image station.

### Dicer cleavage

Wildtype and mutant TAR were digested with recombinant Dicer (Stratagene) according to the manufacturer's directions. After cleavage, RNA was resolved on a 6% TBE-Urea gel and stained with ethidium bromide. Densitometry was performed to verify the presence of cleavage products. For each TAR variant intensity of the undigested input band was set to 100% and the relative intensity of the region of the gel corresponding to 21 nucleotides was determined for both Dicer digested and un-digested lanes.

### RNase protection assay

Total RNA was extracted from HLM-1, OM10.1, ACH2 and HeLa cells treated or untreated with TSA for 24 hours using Trizol reagent (Invitrogen).

Radio-labeled RNA probes were prepared by annealing a DNA oligo containing the T7 promoter sequence to a complementary oligo containing sequence from the 5' portion of TAR (Table 1). Annealed template was transcribed using the MaxiScript T7 transcription kit (Ambion) using α-^32^P ATP. Following transcription the 37 nucleotide RNA probe was purified by separation on a 15% TBE-Urea poly-acrylamide gel (Invitrogen) followed by elution and precipitation of the RNA probe.

Presence of a 21 nucleotide HIV derived miRNA was detected with the RPA III kit (Ambion) as described by the manufacturer. In brief, 30 microgram of total RNA was hybridized to 2 × 10^5 ^CPM of probe. Hybridized products were separated on a 15% TBE-Urea gel. Protected probe was detected by exposure of the gel to a phosphor-imager cassette.

### Luciferase assays

Forty-eight hours after transfection, luciferase activity of renilla and firefly luciferase was assayed with the DualGlo Luciferase Assay (Promega). Luminescence was measured on an EG&G Berthold luminometer. LTR driven firefly luciferase levels were normalized to CMV driven renilla luciferase readings. Data shown represents at least three repeats of each condition.

### Chromatin immunoprecipitation

293 cells were transfected with pLTR-Luc and either siEGFP, siTAR1 or TAR-WT RNA. After 48 hours cells were pelleted, washed, crosslinked with 1% formaldehyde, lysed and sonicated to shear DNA to ~1000 bp. Lysate was immunoprecipitated with 5 micrograms of HDAC-1 antibody (Abcam #ab7028) or a control anti-rabbit IgG. PCR was performed on DNA diluted 1:20 using LTR or luciferase specific primers (Table 1).

To evaluate HDAC recruitment to an integrated HIV-1 LTR TZMbl cells were used. TZMbl were treated for 7 days with TSA to remove HDAC-1, then ChIPed for HDAC-1 LTR after recovery with or without transfection of various RNAs.

## Authors' contributions

ZK carried out the binding assays, cleavage assays, luciferase assays, conceived of the studies and drafted the manuscript. PK carried out the luciferase and western blot assays. MVG, RB and RW carried out the ChIP assays. MH performed the q-RT-PCR. FK participated in the design and coordination of this work and helped draft the manuscript. All authors read and approved the final manuscript.
